# Supplementary material for: Improvements needed to support people living and working with a rare disease in Northern Ireland: current rare disease support perceived as inadequate
Source: Orphanet J Rare Dis. 2020 Nov 9;15:315. doi: 10.1186/s13023-020-01559-6 (PMC7649905; doi:10.1186/s13023-020-01559-6)
Supplement: Supplementary file 1 — Additional file 1. Detailed survey responses. [file 13023_2020_1559_MOESM1_ESM.pdf]

Additional file 2

Detailed survey responses

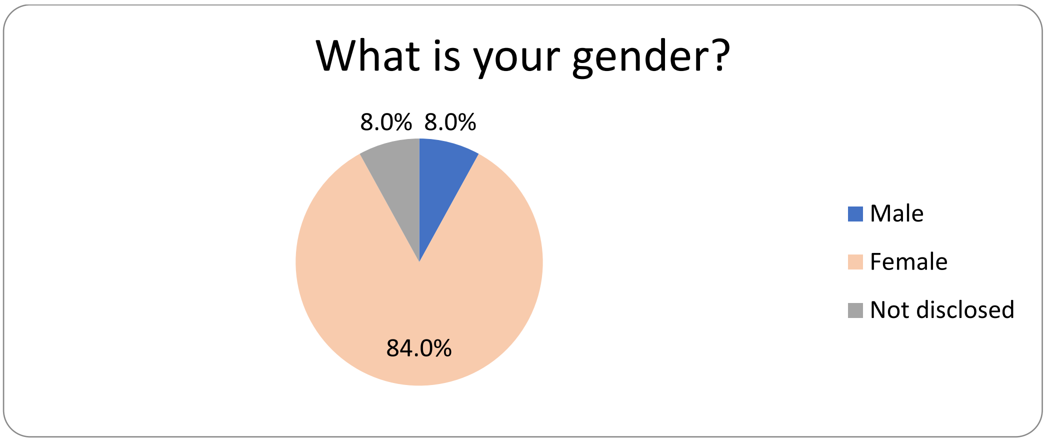

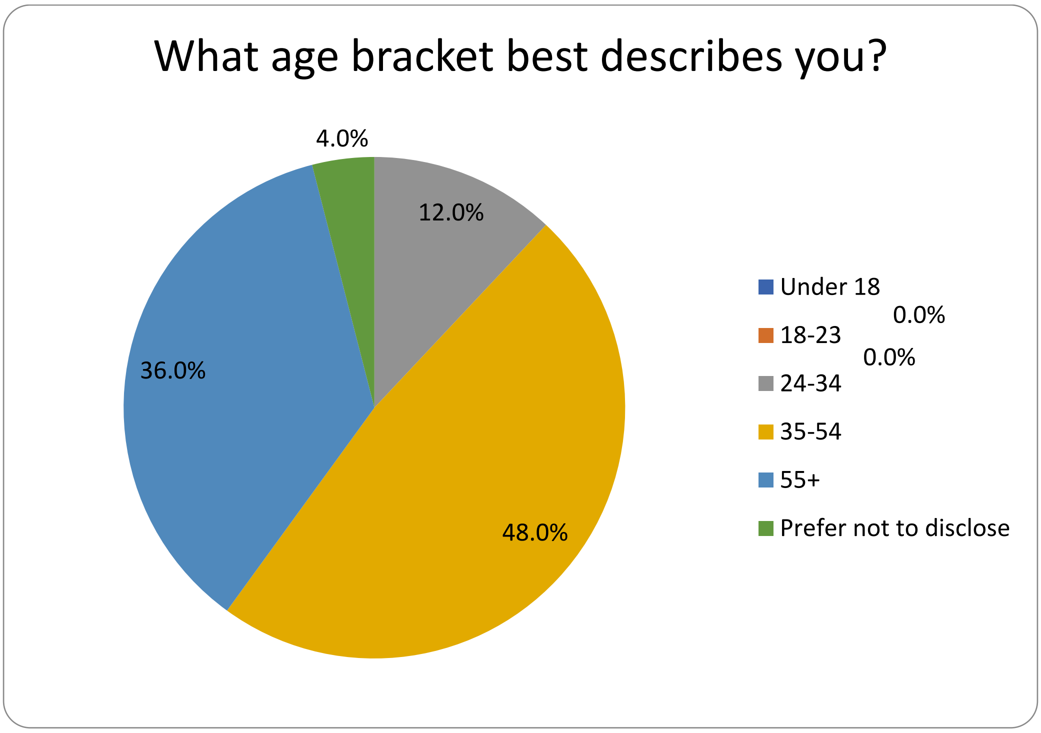

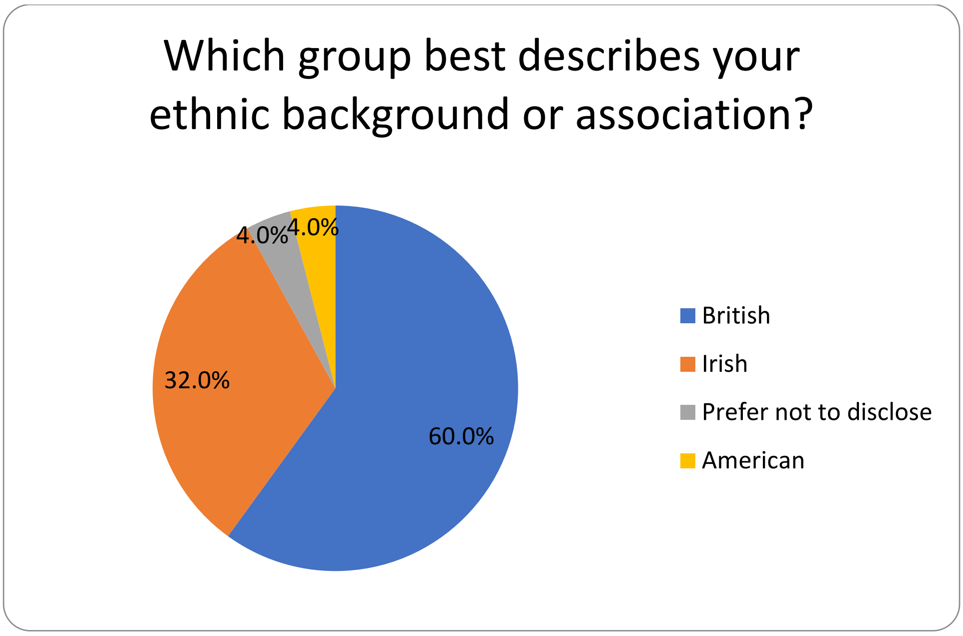
Which group best describes your ethnic background or association?
4.0%
4.0%
32.0%
60.0%
British
Irish
Prefer not to disclose
American

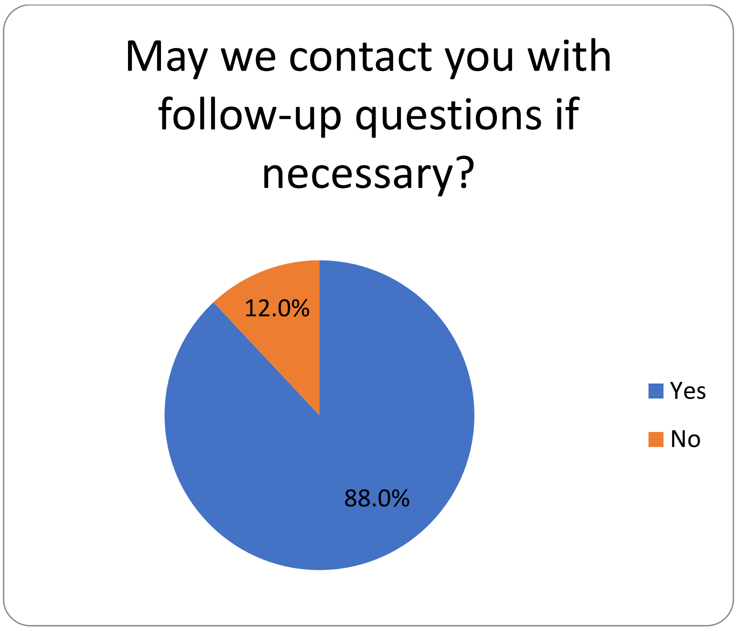
May we contact you with follow-up questions if necessary?
12.0%
88.0%
Yes
No

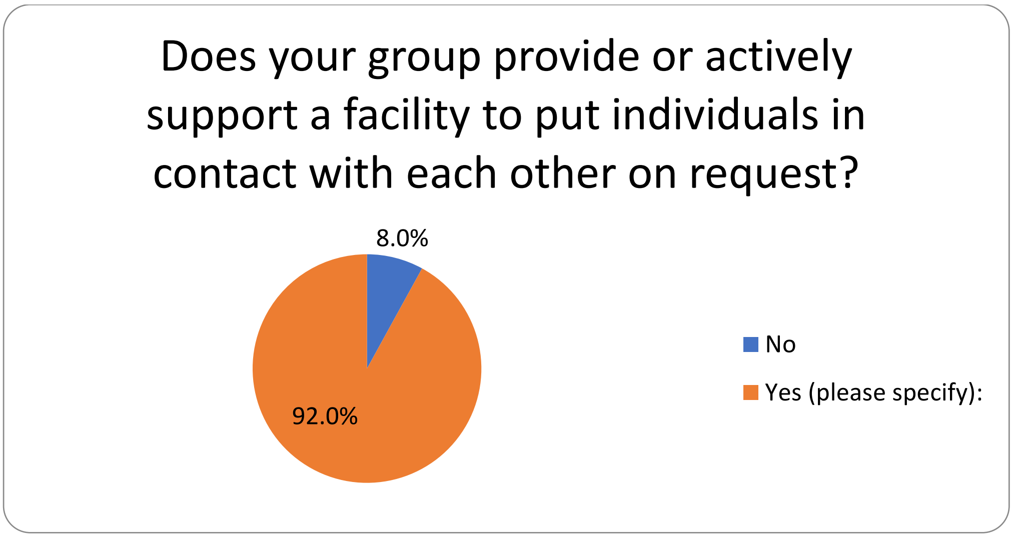
Does your group provide or actively support a facility to put individuals in contact with each other on request?
8.0%
92.0%
No
Yes (please specify):

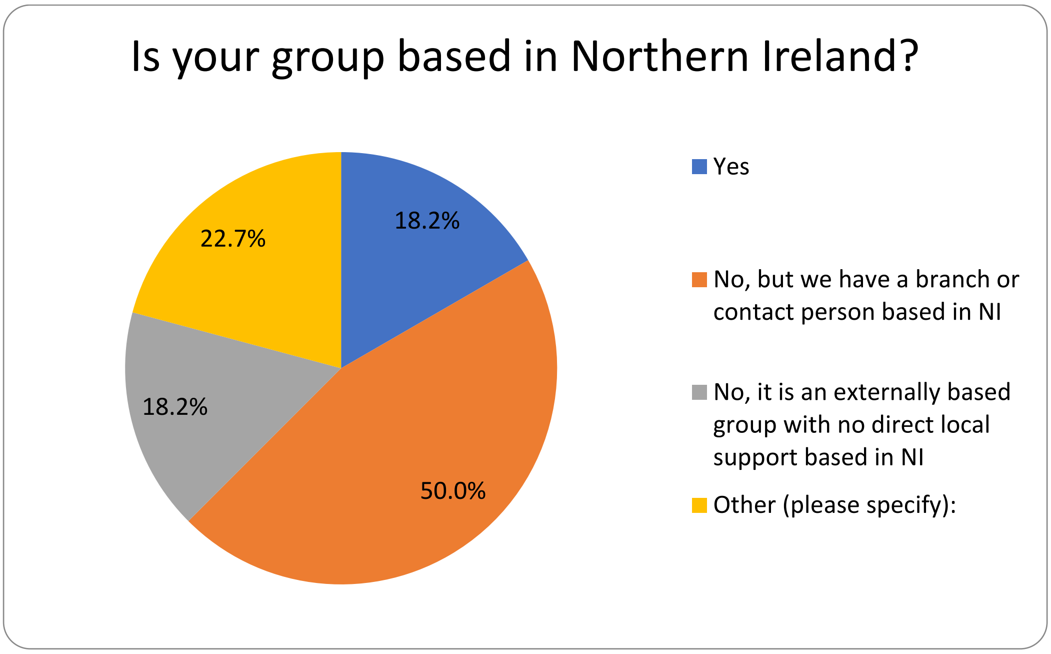
Is your group based in Northern Ireland?
18.2%
50.0%
18.2%
22.7%
Yes
No, but we have a branch or contact person based in NI
No, it is an externally based group with no direct local support based in NI
Other (please specify):

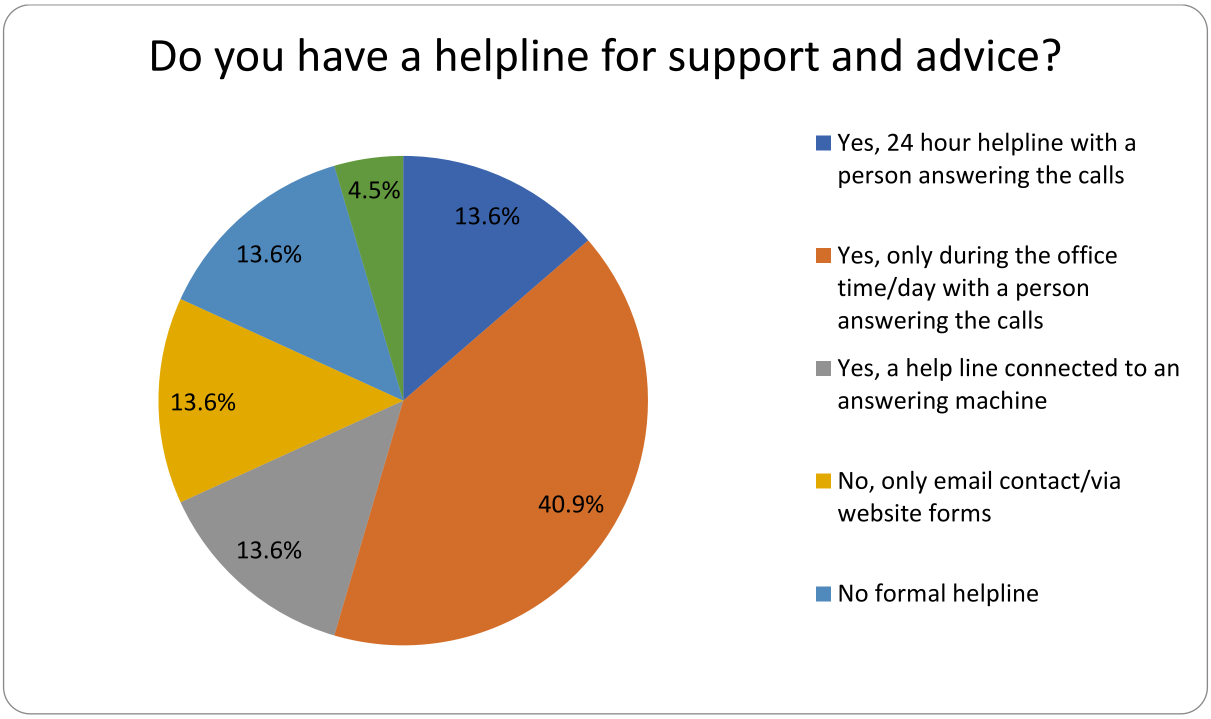
Do you have a helpline for support and advice?
13.6%
40.9%
13.6%
13.6%
13.6%
4.5%
Yes, 24 hour helpline with a person answering the calls
Yes, only during the office time/day with a person answering the calls
Yes, a help line connected to an answering machine
No, only email contact/via website forms
No formal helpline

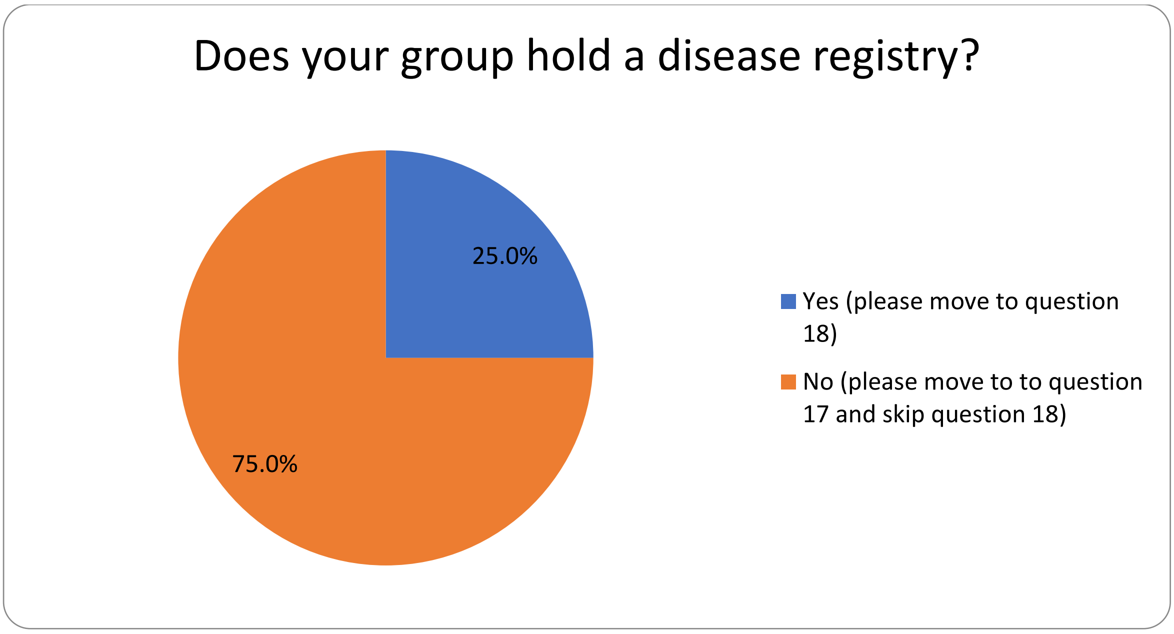
Does your group hold a disease registry?
25.0%
75.0%
Yes (please move to question 18)
No (please move to to question 17 and skip question 18)

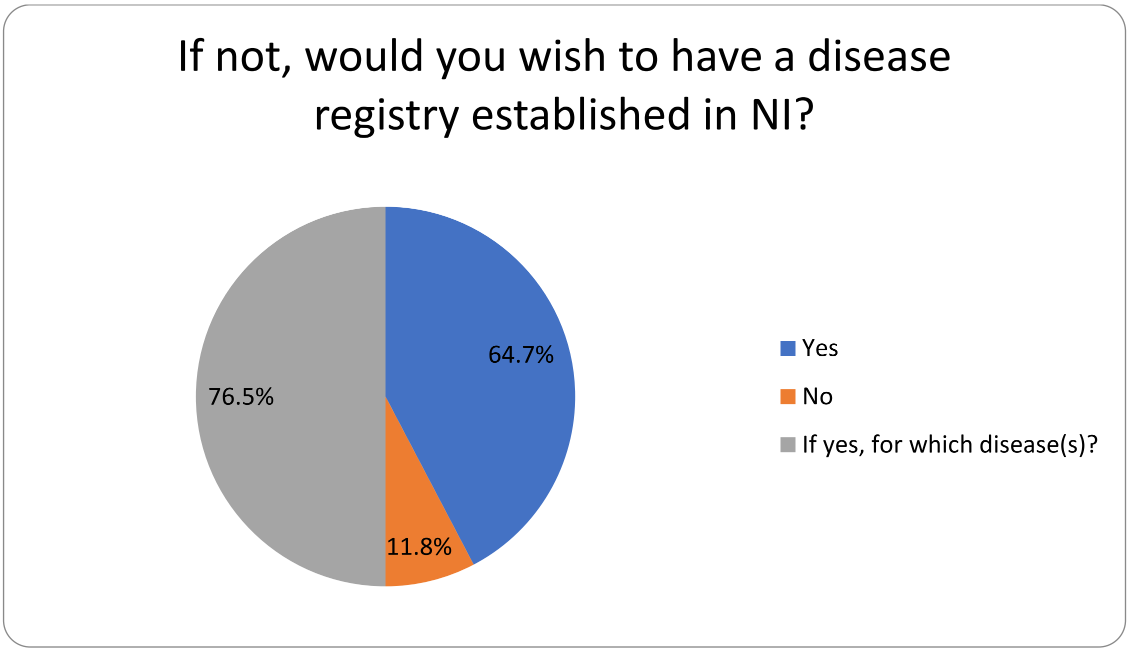
If not, would you wish to have a disease registry established in NI?
64.7%
76.5%
11.8%
Yes
No
If yes, for which disease(s)?

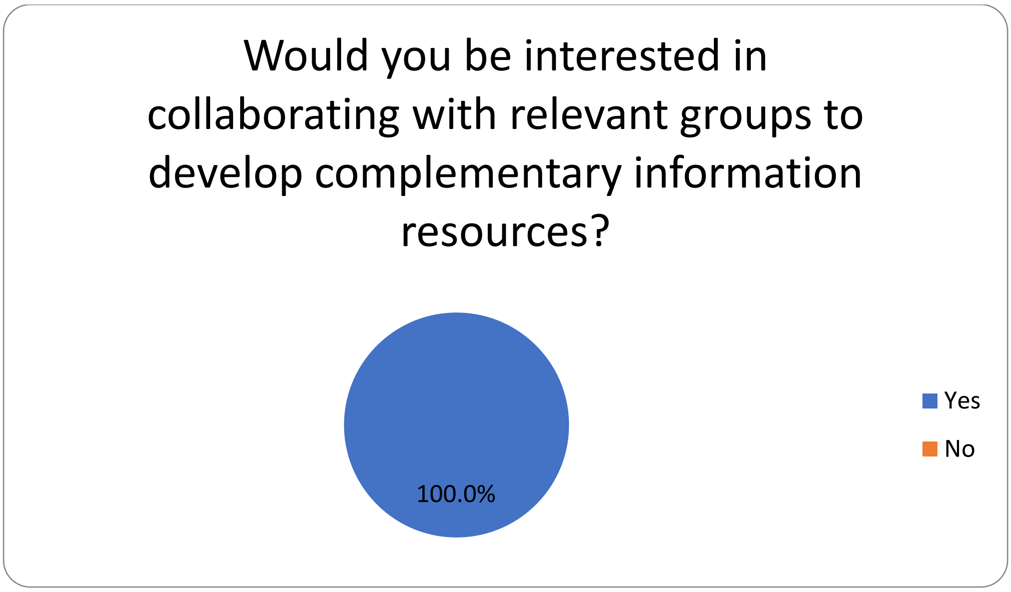
Would you be interested in collaborating with relevant groups to develop complementary information resources?
100.0%
Yes
No

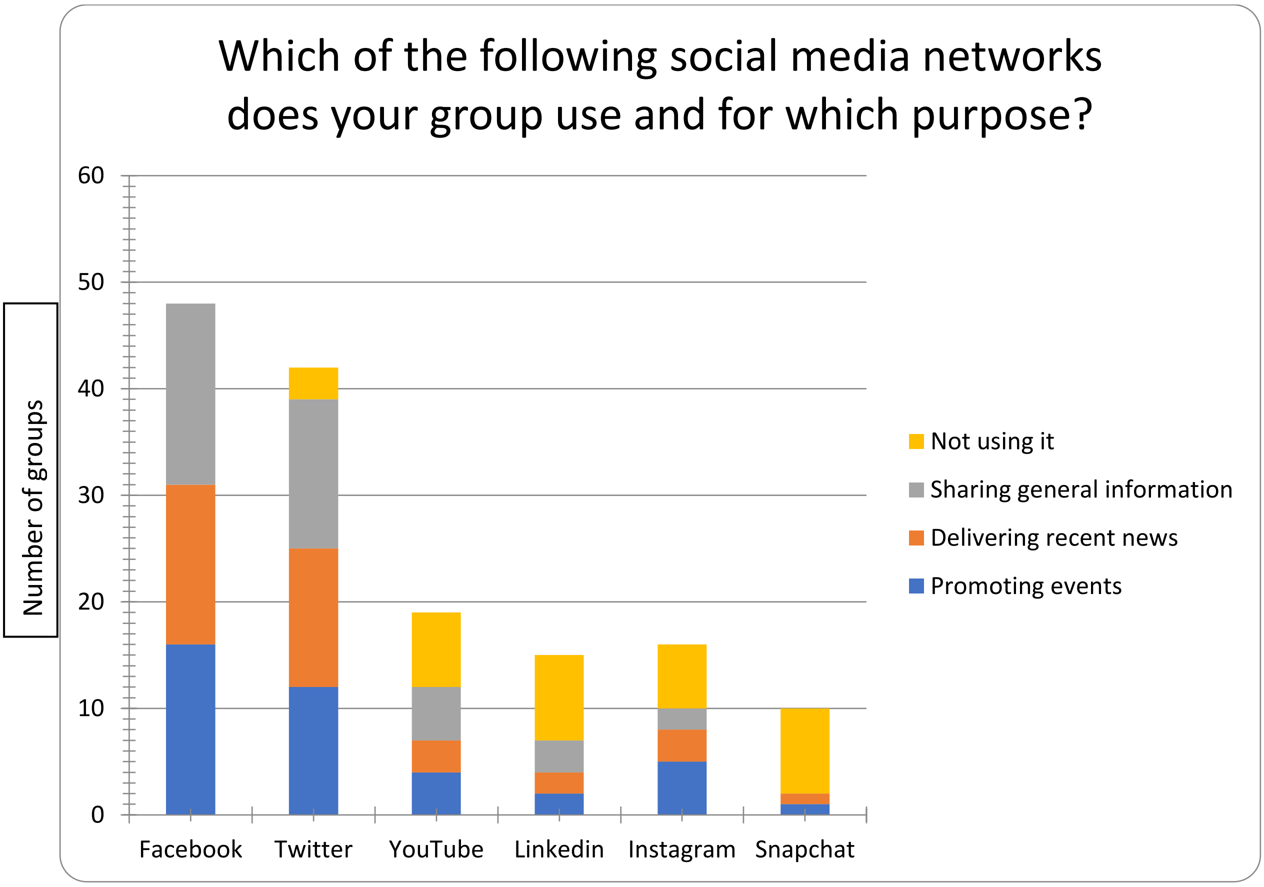
Which of the following social media networks does your group use and for which purpose?
Number of groups
60
50
40
30
20
10
0
Facebook
Twitter
YouTube
Linkedin
Instagram
Snapchat
Not using it
Sharing general information
Delivering recent news
Promoting events

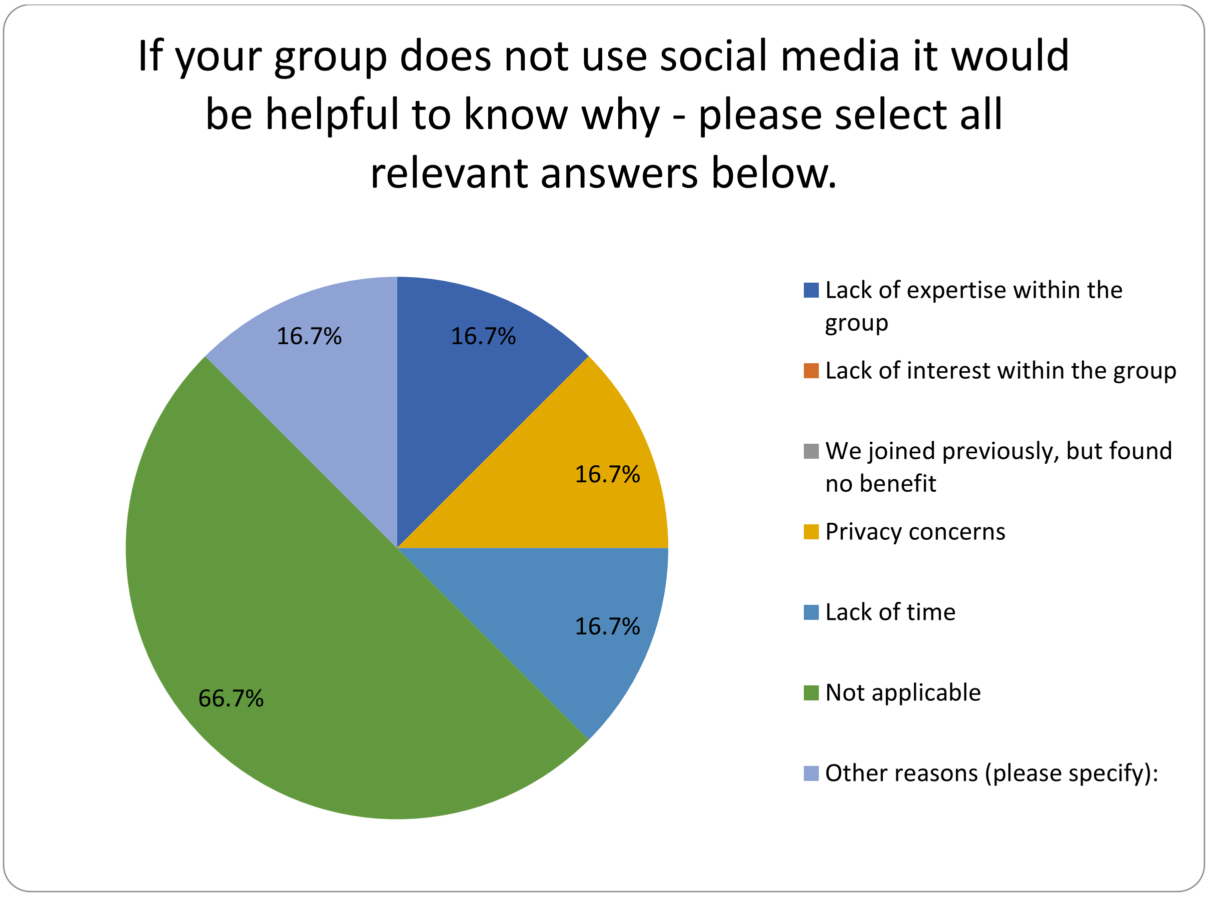
If your group does not use social media it would be helpful to know why - please select all relevant answers below.
16.7%
16.7%
16.7%
16.7%
66.7%
Lack of expertise within the group
Lack of interest within the group
We joined previously, but found no benefit
Privacy concerns
Lack of time
Not applicable
Other reasons (please specify):
