# Supplementary material for: Improvements needed to support people living and working with a rare disease in Northern Ireland: current rare disease support perceived as inadequate
Source: Orphanet J Rare Dis. 2020 Nov 9;15:315. doi: 10.1186/s13023-020-01559-6 (PMC7649905; doi:10.1186/s13023-020-01559-6)
Supplement: Supplementary file 2 — Additional file 2. Survey template. [file 13023_2020_1559_MOESM2_ESM.pdf]

Rare Disease Information from Groups

1. Introduction to Survey

This survey is to help evaluate public awareness of rare diseases, alongside related information and educational resources available for patients, their families, and healthcare professionals in Northern Ireland.

This particular survey is aimed at individual or collaborative groups (this includes a voluntary group, support group, charity etc) and is being conducted as part of the evaluation phase of key priorities for action in the NI Rare Diseases Implementation Plan. Results from this survey will be collated anonymously and used to help improve the accessibility of resources and addressing the needs of individuals affected by rare disease(s) in Northern Ireland. We know many groups have fabulous information resources and are keen to locate existing resources, as well as identify gaps where the development of further resources would be appropriate.

Please do indicate if you are happy to be contacted (question 4) should there be follow-up questions. We appreciate and welcome your input to this evaluation. If you wish to provide further details and / or have any questions about this survey, please contact julie.mcmullan@qub.ac.uk using ‘Survey groups' in the subject line.

It is not essential to answer every question! Complete survey data is most useful to us, but we understand you may not wish to disclose (or have time to complete) some information – please feel free to skip over any such question(s).

Many thanks for your participation; it is much appreciated!

2. General Information

We ask this information to help evaluate if we have responses from a diverse group of individuals; we are keen to maximise inclusion! These details are about the respondent/representative of the group.

1. What is your gender?

- ☐ Male
- ☐ Female
- ☐ Prefer not to disclose
- ☐ Prefer to identify as:

2. What age bracket best describes you?

- ☐ Under 18
- ☐ 18-23
- ☐ 24-34
- ☐ 35-54
- ☐ 55+
- ☐ Prefer not to disclose

3. Which group best describes your ethnic background or association?

- ☐ British
- ☐ Irish
- ☐ Asian
- ☐ African American
- ☐ Prefer not to disclose
- ☐ Other (please specify):

4. May we contact you with follow-up questions if necessary?

- [ ] Yes
- [ ] No

If yes, please include the preferred contact details below:

3. Group Information Basics

Please tell us a little about your group.

5. What is the name of your group?

6. Please tell us if your group has been known by other names.

7. Which rare disease(s) does your group focus on?

8. Does your group provide or actively support a facility to put individuals in contact with each other on request e.g. healthcare professionals, patients, families, carers through a network facility such as an online forum / discussion groups, sharing contact details etc?

- [ ] No
- [ ] Yes (please specify):

4. Group Information Details

9. How and when was your group established. Please let us know if it is a company Ltd by guarantee, a registered Charity in the UK, NI, ROI etc.

10. Do you have an information or communication manager?

- [ ] Yes
- [ ] No

If yes, please provide their name and contact details:

11. Is your group based in Northern Ireland?

- [ ] Yes
- [ ] No, but we have a branch or contact person based in NI
- [ ] No, it is an externally based group with no direct local support based in NI
- [ ] Other (please specify):

Our head office is based in:

12. Do you have a helpline for support and advice?

- [ ] Yes, 24 hour helpline with a person answering the calls
- [ ] Yes, only during the office time/day with a person answering the calls
- [ ] Yes, a help line connected to an answering machine
- [ ] No, only email contact/via website forms
- [ ] No formal helpline
- [ ] Non-urgent information line contact only

13. Which of the following (if any) specific types of events does your group organise?

- [ ] Fundraising events
- [ ] Meet and greet between individuals living with rare disease(s)
- [ ] Meeting between health professionals and patients/families
- [ ] Raising awareness days
- [ ] Research days
- [ ] Training days
- [ ] Other (please specify):

14. Does your group hold a list of members?

- [ ] Yes
- [ ] No

If yes, it would be useful to learn about what information you collect and find most useful:

15. Will the expected data protection regulations cause challenges for your group?

- [ ] Yes
- [ ] No
- [ ] Unaware of these regulations
- [ ] The impact is not yet known

If yes, what do you anticipate these challenges to be?

5. Information About Registries

This page is intended to help update information from our 2014 survey of resources available to individuals living in NI. If you prefer to speak to one of our team directly, please do get in touch with Dr Julie McMullan at julie.mcmullan@qub.ac.uk

16. Does your group hold a disease registry?

- [ ] Yes (please move to question 18)
- [ ] No (please move to to question 17 and skip question 18)

17. If not, would you wish to have a disease registry established in NI?

- [ ] Yes
- [ ] No
- [ ] If yes, for which disease(s)?

18. Please tell us a little about the registry, for example does it include:

	Yes	No
Patient submitted data	☐	☐
Healthcare personnel submitted data (Doctor, allied health professional etc.)	☐	☐
Natural history of disease over time (i.e. longitudinal data for individuals)	☐	☐
Available clinical trials	☐	☐
Emerging therapies	☐	☐
Links to national resources	☐	☐
Links to international resources	☐	☐

Please let us know the funding mechanism for the registry(s) - is this long-term funding for the future?

6. About Your Website

19. How often do you update your website?

- [ ] Daily
- [ ] Weekly
- [ ] Monthly
- [ ] Quarterly
- [ ] Annually
- [ ] As soon as a new piece of information is available
- [ ] Other (please specify):

20. On your website, do you offer any specific information about any of these topics:This question is repeated at the end for anyone wishing to enter details more than once per row...

	For healthcare professionals	For younger patients (<18 years)	For adult patients	For carers	Would like to develop	No	Not relevant
Current research and discoveries	☐	☐	☐	☐	☐	☐	☐
Current treatment options	☐	☐	☐	☐	☐	☐	☐
Diagnostic approaches	☐	☐	☐	☐	☐	☐	☐
Financial / social / educational support links	☐	☐	☐	☐	☐	☐	☐
Flyer for individual disease(s)	☐	☐	☐	☐	☐	☐	☐
Information pack at key stages (e.g. newly diagnosed)	☐	☐	☐	☐	☐	☐	☐
Inheritance facts	☐	☐	☐	☐	☐	☐	☐
Mechanisms of the rare disease(s)	☐	☐	☐	☐	☐	☐	☐
Medications and side effects	☐	☐	☐	☐	☐	☐	☐
Network of key services (consultants, physiotherapy sites, accessible swimming pools etc)	☐	☐	☐	☐	☐	☐	☐
Nutritional advice	☐	☐	☐	☐	☐	☐	☐
Online Discussion forum	☐	☐	☐	☐	☐	☐	☐
Prognosis and outcomes	☐	☐	☐	☐	☐	☐	☐
Research funding links	☐	☐	☐	☐	☐	☐	☐
Training / educational events	☐	☐	☐	☐	☐	☐	☐

Others (please explain):

21. Apart from the groups mentioned above, who else do you believe can benefit from your website?

22. Does your website have a visitor counter?

- [ ] Yes
- [ ] No

if yes, on average how many times a day is your website consulted?

7. Further Information Resources

23. Do you have information/communication strategies under development?

- [ ] No
- [ ] Yes (please describe):

24. Does the information you provide have a quality mark for formal information standards (e.g. http://www.england.nhs.uk/tis/)

- [ ] No
- [ ] Yes

If yes, which organisation evaluates / approves / assures the information?

25. Has your group developed/coordinated training modules for healthcare professionals?

- [ ] No
- [ ] Yes (please describe):

26. Has your group developed/coordinated any smartphone-based apps?

- [ ] No
- [ ] Yes (please describe):

8. Interacting with Your Resources

27. How would you wish those living with rare diseases in NI to primarily interact with your information resources?

28. How would you wish healthcare professionals to primarily interact with your information resources?

29. How would you wish non-clinical professionals to primarily interact with your information resources?

30. How would you wish other groups in NI to primarily interact with your information resources?

31. In an ideal world with unlimited funding, how should information and communication for rare diseases be improved in NI inside your team?

32. In an ideal world with unlimited funding, how should information and communication for rare diseases be improved in NI outside your group?

33. Would you be interested in collaborating with relevant groups to develop complementary information resources?

- [ ] Yes
- [ ] No

34. Please tell us any additional information you feel would be useful to consider:

9. Social media links

We are very keen to learn how groups interact with social media - if you use it, what works, what doesn't, which resources do you find most helpful for which reasons...

35. Which of the following social media networks does your group use and for which purpose?

	Promoting events	Delivering recent news	Sharing general information	Not using it
Facebook	☐	☐	☐	☐
Twitter	☐	☐	☐	☐
YouTube	☐	☐	☐	☐
Linkedin	☐	☐	☐	☐
Instagram	☐	☐	☐	☐
Snapchat	☐	☐	☐	☐

Other social media purposes (please explain):

36. If your group does not use social media it would be helpful to know why - please select all relevant answers below.

- ☐ Lack of expertise within the group
- ☐ Lack of interest within the group
- ☐ We joined previously, but found no benefit
- ☐ Privacy concerns
- ☐ Lack of time
- ☐ Not applicable
- ☐ Other reasons (please specify):

37. For what purpose does your group use social media? Tick all that apply.

- ☐ Communication with other organisations
- ☐ Hosting support networks
- ☐ Posting information about new clinical trials
- ☐ Promoting fundraising campaigns
- ☐ Raising public awareness of rare diseases
- ☐ Sharing information about upcoming events
- ☐ Sharing information about your specific rare disease
- ☐ Sharing recent news
- ☐ This group does not use social media
- ☐ Other reasons (please specify):

38. How regularly does your group post material on social media?

- ☐ Daily
- ☐ Weekly
- ☐ Monthly
- ☐ Quarterly
- ☐ Annually
- ☐ When new information is released
- ☐ I don't know
- ☐ This group does not use social media
- ☐ Other (please specify):

39. Does your group have a dedicated representative(s) responsible for maintaining the social media account(s)?

- [ ] Yes
- [ ] No
- [ ] I don't know

40. How many followers/members does your group have in the following social networks?

	None	1-99	100-499	500-999	1000-5000	5000+	I don't know
Facebook	☐	☐	☐	☐	☐	☐	☐
Twitter	☐	☐	☐	☐	☐	☐	☐
YouTube	☐	☐	☐	☐	☐	☐	☐
Instagram	☐	☐	☐	☐	☐	☐	☐

Additional social media numbers

41. Please indicate your level of agreement with the statement listed below:Social media use by rare disease groups is beneficial for communication with, and support for, patients.

- [ ] Strongly agree
- [ ] Agree
- [ ] Slightly agree
- [ ] Neutral
- [ ] Slightly disagree
- [ ] Disagree
- [ ] Strongly disagree

42. Do you think that your group should expand its use of social media given unlimited time and budget?

- [ ] Yes
- [ ] No
- [ ] No opinion

If yes, what would be the first thing you'd like to do:

43. Repeat of question 20, allowing multiple answers per row.On your website, do you offer any specific information about any of these topics:

	For healthcare professionals	For younger patients (<18 years)	For adult patients	For carers	Would like to develop	No	Not relevant
Current research and discoveries	☐	☐	☐	☐	☐	☐	☐
Current treatment options	☐	☐	☐	☐	☐	☐	☐
Diagnostic approaches	☐	☐	☐	☐	☐	☐	☐
Financial / social / educational support links	☐	☐	☐	☐	☐	☐	☐
Flyer for individual disease(s)	☐	☐	☐	☐	☐	☐	☐
Information pack at key stages (e.g. newly diagnosed)	☐	☐	☐	☐	☐	☐	☐
Inheritance facts	☐	☐	☐	☐	☐	☐	☐
Mechanisms of the rare disease(s)	☐	☐	☐	☐	☐	☐	☐
Medications and side effects	☐	☐	☐	☐	☐	☐	☐
Network of key services (consultants, physiotherapy sites, accessible swimming pools etc)	☐	☐	☐	☐	☐	☐	☐
Nutritional advice	☐	☐	☐	☐	☐	☐	☐
Online Discussion forum	☐	☐	☐	☐	☐	☐	☐
Prognosis and outcomes	☐	☐	☐	☐	☐	☐	☐
Research funding links	☐	☐	☐	☐	☐	☐	☐
Training / educational events	☐	☐	☐	☐	☐	☐	☐

Others (please explain):
