# Supplementary material for: Improvements needed to support people living and working with a rare disease in Northern Ireland: current rare disease support perceived as inadequate
Source: Orphanet J Rare Dis. 2020 Nov 9;15:315. doi: 10.1186/s13023-020-01559-6 (PMC7649905; doi:10.1186/s13023-020-01559-6)
Supplement: Supplementary file 3 — Additional file 3. Supportive networking options provided by rare disease collaborative groups to help connect people. [file 13023_2020_1559_MOESM3_ESM.pdf]

Additional file 3

Supportive networking options provided by rare disease collaborative groups to help connect people.

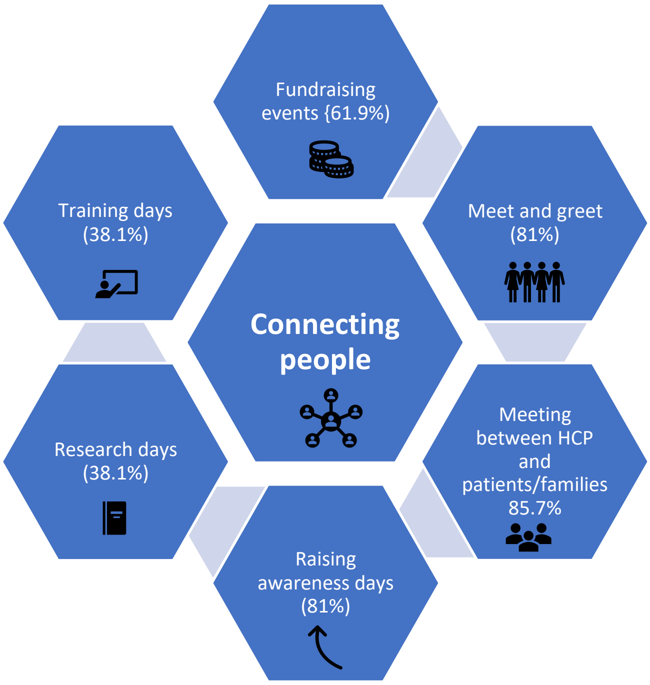

HCP – health care personnel
