# Supplementary material for: Improvements needed to support people living and working with a rare disease in Northern Ireland: current rare disease support perceived as inadequate
Source: Orphanet J Rare Dis. 2020 Nov 9;15:315. doi: 10.1186/s13023-020-01559-6 (PMC7649905; doi:10.1186/s13023-020-01559-6)

Additional file 4

Top purposes of social media platforms and most popular information offered on websites.

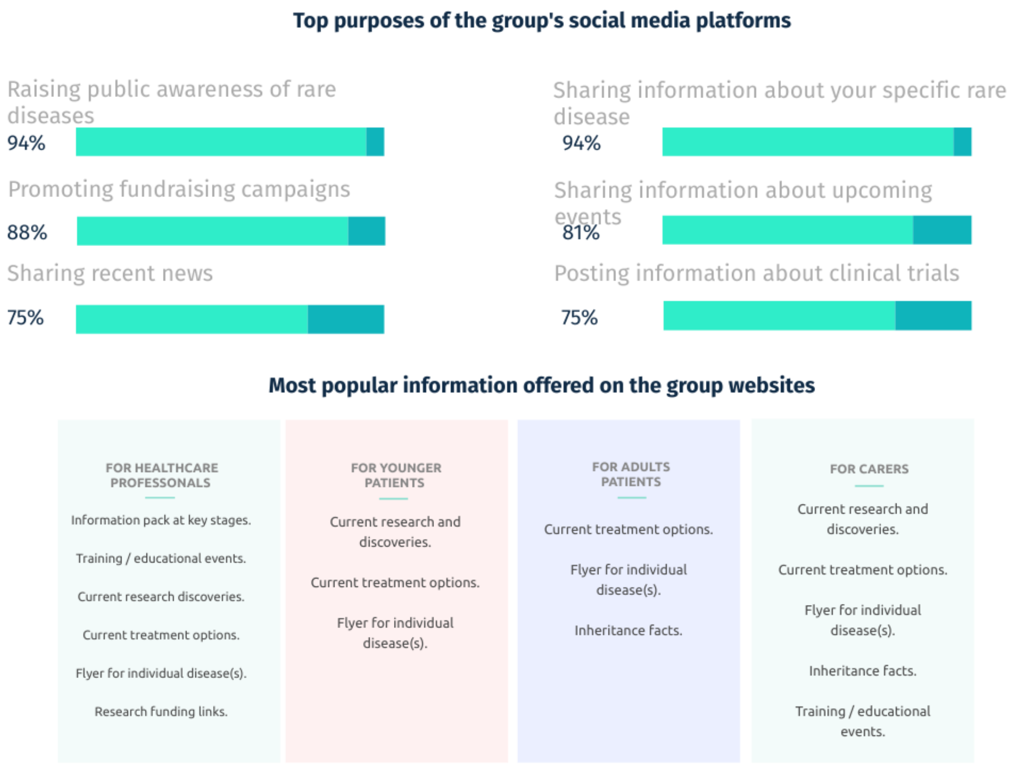

Supplement: Supplementary file 4 — Additional file 4. Top purposes of social media platforms and most popular information offered on websites. [file 13023_2020_1559_MOESM4_ESM.pdf]
