# Supplementary figures and images for: Improvements needed to support people living and working with a rare disease in Northern Ireland: current rare disease support perceived as inadequate
Source: Orphanet J Rare Dis. 2020 Nov 9;15:315. doi: 10.1186/s13023-020-01559-6 (PMC7649905; doi:10.1186/s13023-020-01559-6)

## Additional file 5

### Social media use by rare disease collaborative groups

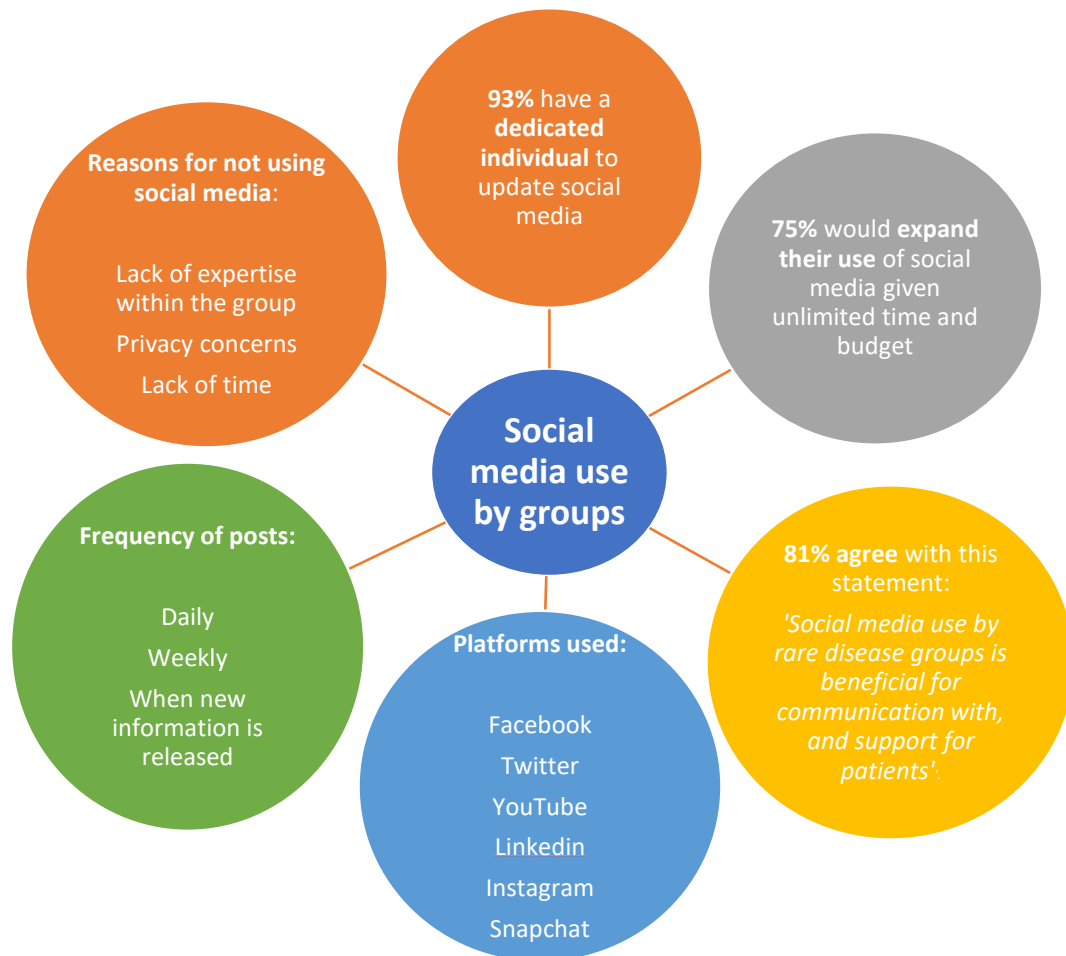

Supplement: Supplementary file 5 — Additional file 5. Social media use by rare disease collaborative groups. [file 13023_2020_1559_MOESM5_ESM.pdf]

## Additional file 6

### Distribution of 'registry' data

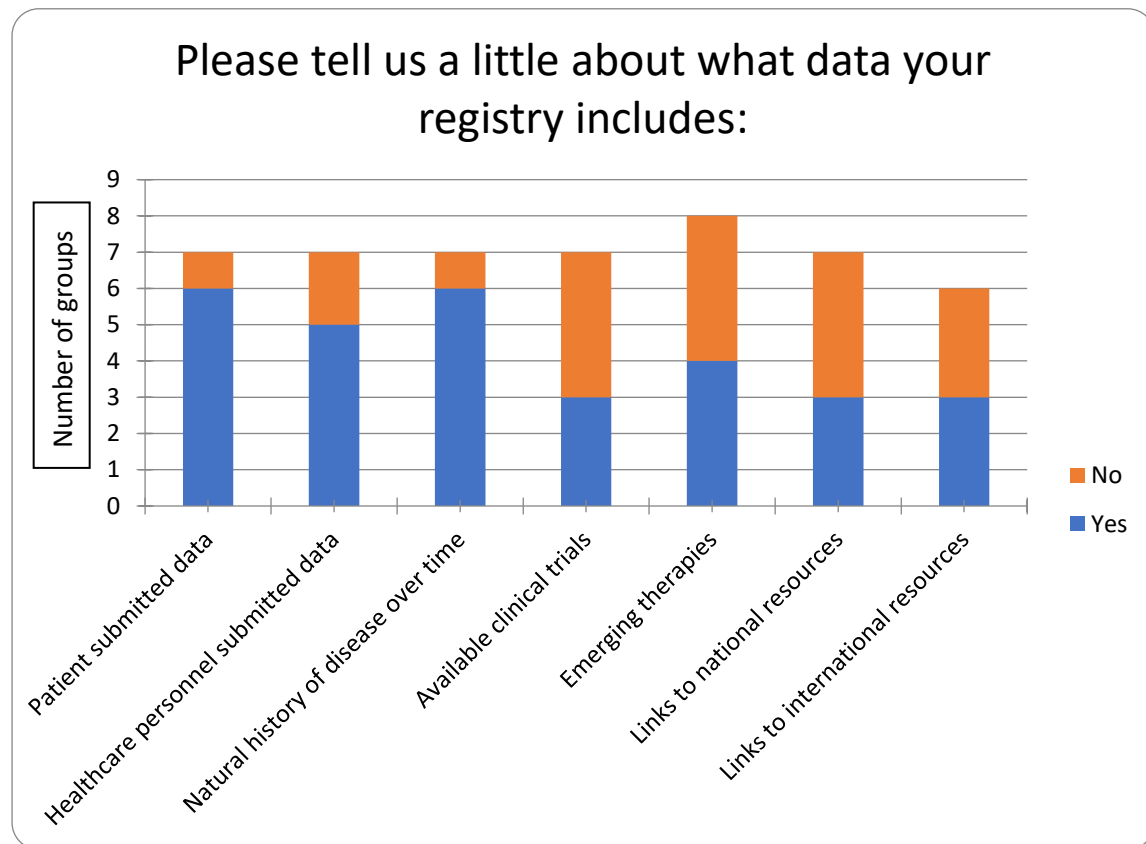

Supplement: Supplementary file 6 — Additional file 6. Distribution of ‘registry’ data. [file 13023_2020_1559_MOESM6_ESM.pdf]

## Additional file 9

### Recommendations to inform health care policy and practice

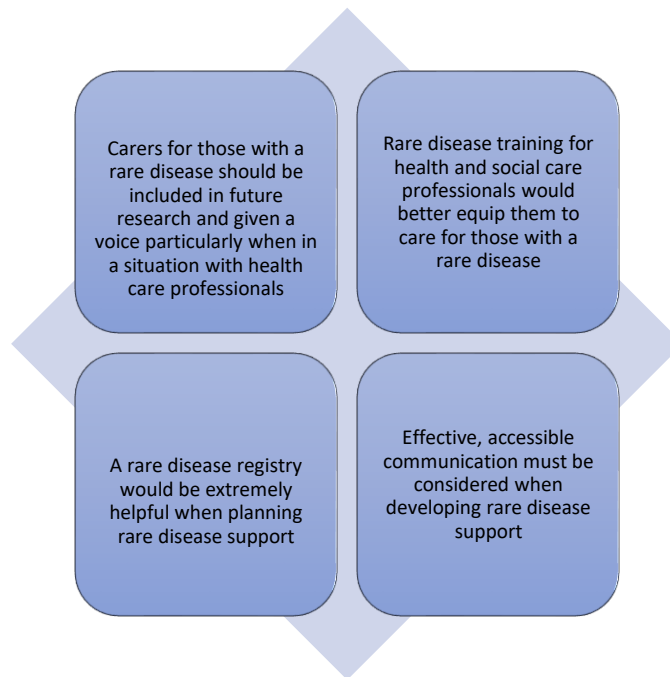

Supplement: Supplementary file 9 — Additional file 9. Recommendations to inform health care policy and practice. [file 13023_2020_1559_MOESM9_ESM.pdf]
