# Supplementary material for: Improvements needed to support people living and working with a rare disease in Northern Ireland: current rare disease support perceived as inadequate
Source: Orphanet J Rare Dis. 2020 Nov 9;15:315. doi: 10.1186/s13023-020-01559-6 (PMC7649905; doi:10.1186/s13023-020-01559-6)
Supplement: Supplementary file 7 — Additional file 7. How information and communication could be improved within and beyond rare disease collaborative groups. [file 13023_2020_1559_MOESM7_ESM.pdf]

Additional file 7

How information and communication could be improved within and beyond rare disease collaborative groups.

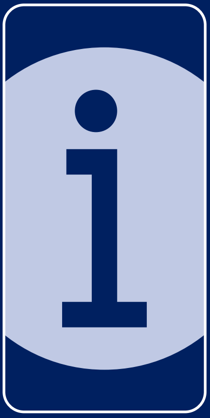

INSIDE THE GROUPS

- Supporting a local person to get involved - e.g. a local ambassador.
- More staff.
- Hosting conferences and / or information days.
- Tailor information to country-specific legislation.
- Free database for all rare genetic conditions with appropriate links.
- Keeping staff well informed of services and improvements.
- Facilitiating familes and HCPs to co-develop services.
- Face to face meetings of Patient Representative Panel(s).

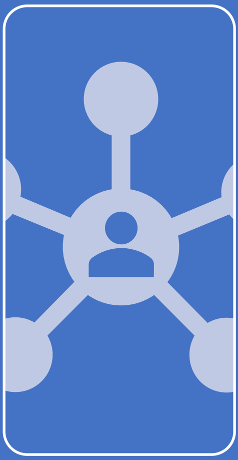

OUTSIDE THE GROUPS

- Doctors recommending RD patients to contact a collaborative group.
- Better sharing of information between groups; collaborative website
- Access to information for therapies, aids, grants and clinical trials
- More collaboration on raising awareness and policy.
- Using social media to disseminate 'personal stories'.
- Creation of a local 'registry' for all rare conditions
- Developing a 'rare disease' ECHO project for HCPs
- Improved communication between GPs and other health persons
- Making educational visits to schools and workplaces.
- Involvement in international events.
- Having a rare disease communication officer.
- Holding bi-annual meetings and awareness days.
- Regular workshops with roundtable discussions.

HCP: Health care personnel

RD: Rare disease

ECHO: Extension for Community Healthcare Outcomes
