# Supplementary material for: Improvements needed to support people living and working with a rare disease in Northern Ireland: current rare disease support perceived as inadequate
Source: Orphanet J Rare Dis. 2020 Nov 9;15:315. doi: 10.1186/s13023-020-01559-6 (PMC7649905; doi:10.1186/s13023-020-01559-6)
Supplement: Supplementary file 8 — Additional file 8. Summary table of topics raised in the discussion. [file 13023_2020_1559_MOESM8_ESM.pdf]

Additional file 8: Summary table of topics raised in the discussion

**Communication is an important factor to consider when developing rare disease support**	
Communication within the health service	Improved communication between General Practitioners (GP) and other healthcare professionals is required, especially at diagnosis.
Communication within rare disease collaborative groups	A move is happening away from face to face support to more support *via* online platforms, but with this change effectiveness of support provided needs to be evaluated.
Online communication	Use of online platforms is dependent on the habits and preferences of individuals. More investment in the online presence of groups may allow for more people to be supported.
Collaboration	Collaboration between groups is valued, but also collaboration between healthcare professionals, and then between the groups and healthcare professionals.

**Rare disease registries**	There is a desire from the majority of groups in the study for a rare disease registry in Northern Ireland which would help them to be more responsive in their support and advocating for service planning for rare disease patients.
**Training**	Some training is currently provided for healthcare professionals by the groups but evidence shows that more is required to improve the support provided to those with a rare disease, and understanding within the healthcare system.
**Carers**	The groups were very aware that better support is needed for carers of those with a rare disease, with these carers often being overlooked. Further research on how best to provide this support is needed.
**Current care/support for those with a rare disease is not adequate.**	Finding and securing funding to improve and develop the services available in Northern Ireland was perceived as important. The collaborative groups had many suggestions on how to improve these

	services and should be consulted.
